# Effect of resisted exercise on autonomic cardiac modulation in elderly women: STROBE-compliant study

**DOI:** 10.1097/MD.0000000000028977

**Published:** 2022-03-11

**Authors:** Naerton José Xavier Isidoro, Fernando Rocha Oliveira, Rodrigo Daminello Raimundo

**Affiliations:** a Universidade Regional do Cariri, Crato, Ceará, Brazil; b Laboratório de Delineamento de Estudos e Escrita Científica, Centro Universitário FMABC, Faculdade de Medicina do ABC, Santo André, SP, Brazil.

**Keywords:** elderly, heart rate variability, resistance exercise

## Abstract

Heart rate variability (HRV) is an important cardiac health marker, with lower values indicating a reduction in vagal control of cardiac rhythm and decreasing significantly with advancing age. In this study, we evaluated the effects of strength exercises for the upper and lower limbs on cardiac autonomic modulation in elderly women.

We registered 29 participants using a portable RS800CX heart rate monitor to record the RR intervals. For the collection of HRV data, the volunteers remained seated at rest for 10 minutes. After the rest period, the participants performed the exercises. Immediately after the exercise protocol, the subjects remained seated at rest for 30 minutes. HRV indices were analyzed in the following periods: rest, 0 to 10 minutes, 5 to 10 minutes, 10 to 20 minutes, and 20 to 30 minutes. Systolic arterial pressure and diastolic arterial pressure were measured in the following periods: rest, immediately after exercise, and 30 minutes after exercise.

Regarding the mean of the RR intervals, heart rate, and indexes of the time and frequency domains surveyed, there were no statistically significant differences between the 4 moments in the protocols for upper and lower limbs. No significant differences were found in systolic and diastolic pressures between the 3 time points surveyed in the protocols for the upper and lower limbs.

Resistance exercises performed with low-intensity loads and a greater number of repetitions did not promote significant variations in cardiac autonomic modulation and blood pressure levels, showing good safety in elderly women.

## 1 Introduction

Aging is a natural, complex, individualized, and irreversible process that affects the biological, psychological, and social spheres of human beings. It is marked by changes in the metabolism and physicochemical properties of cells, triggering structural and functional changes in tissues and organs and influencing the longevity and health of individuals. It is not possible to reverse aging, but strategies based on the adoption of a healthy lifestyle associated with regular physical exercise and a balanced diet can delay its deleterious effects with positive repercussions on the physical fitness of populations, that is, on the ability to perform activities of daily living with willingness and efficiency.^[1,2]^

This phenomenon is characterized by the progressive loss of physiological integrity, which is evidenced as the main risk factor for the incidence of diseases such as cancer, diabetes, cardiovascular disorders, and neurodegenerative diseases.^[3]^

Aging is associated with a considerable decrease in the skeletal muscle mass. This progressive phenomenon, known as sarcopenia, negatively affects individuals and decreases the functional capacity of the elderly.^[4]^

The reduction in muscle mass is associated with aging, increased catabolic processes, and a sedentary lifestyle, contributing to the development of frailty and decreased functional capacity with advancing age. Sarcopenic elderly individuals have a lower parasympathetic-associated modulation.^[4,5]^

Sarcopenia associated with advancing age may accelerate due to factors such as changes in the hormonal environment, inactivity, poor nutrition, chronic disease, and loss of integrity and function in the peripheral and central nervous systems.^[6]^

Strength training has been shown to increase muscle mass, reduce fat percentage, and maintain blood pressure and heart rate at acceptable levels for age.^[7]^

One of the ways to assess the autonomic modulation of heart rate in a noninvasive manner in humans is heart rate variability (HRV), a term conventionally accepted to describe the fluctuations in the intervals between consecutive heartbeats (RR intervals), which are related to the influence of the autonomic nervous system over the sinus node (HRV: standards of measurement, physiological interpretation, and clinical use.^[6,8]^

HRV is an important cardiac health marker, with lower values indicating a reduction in the vagal control of cardiac rhythm. HRV decreases significantly with advancing age, with a greater impact on this reduction among women.^[8]^

HRV in the practice of physical exercise enables the monitoring of cardiovascular fitness and prevention of excessive physical training and postexercise fatigue.^[9–11]^

Autonomic dysregulation, represented by reduced HRV, in experimental and clinical studies, is an important indicator of morbidity and mortality from cardiovascular diseases. Excessive sympathetic tone and/or decreased parasympathetic tone are associated with the risk of coronary events in healthy individuals and is much more pronounced in cardiac patients.^[12]^

Autonomic balance and HRV differ between men and women. Women have a lower sympathetic response than men, a fact that can be cardioprotective with a positive impact on recovery after high-intensity exercise.^[11,13]^

Studies point to an acceleration of lean tissue loss after menopause due to acute changes in estrogen availability and effectiveness. During this period, it is interesting to focus on interventions that seek to alleviate muscle loss in women.^[14]^

Resistance exercises performed with a high number of repetitions and low intensity have a positive influence on muscle strength and endurance over time, as well as beneficially affecting cardiac autonomic regulation at rest. High-intensity resistance exercises, however, promote greater postexercise hypotension and an increase in the vasodilator response and sympathovagal balance in hypertensive women.^[15]^

There are a small number of studies in the literature on resistance exercises for the elderly and their acute effects on cardiac autonomic modulation, therefore needing to expand the investigations on the subject using different training protocols. We hypothesized that low-intensity resistance exercises for the upper and lower limbs in elderly women alter the autonomic modulation of heart rate, demonstrating the safety of these activities in relation to possible cardiovascular risks.

In this way, the present study has as general objective to evaluate the safety and magnitude of the heart rate autonomic response after low-intensity resistance exercise sessions for upper and lower limbs in elderly women, and the specific objectives are as follows:

1. Describe the linear indices in the pre- and post-training periods in resistance training sessions for upper and lower limbs
2. Compare autonomic responses in pre- and post-training periods.

## 2 Method

This cross-sectional study was conducted between March 2019 and March 2020 in Crato, Ceará, Brazil. The study was approved by the Ethics Committee of Universidade Regional do Cariri (No. 1.534.428), and written informed consent was obtained from all participants. Ethical standards were maintained during the study in accordance with CNS Resolution No. 466/12.

### 2.1 Participants

The convenience sample consisted of 29 elderly women who attended the facilities of the fitness center at Cariri University. The inclusion criteria were female individuals aged over 60 years and with a minimum frequency of 2 weekly exercise sessions lasting 50 minutes.

**Figure 1:**
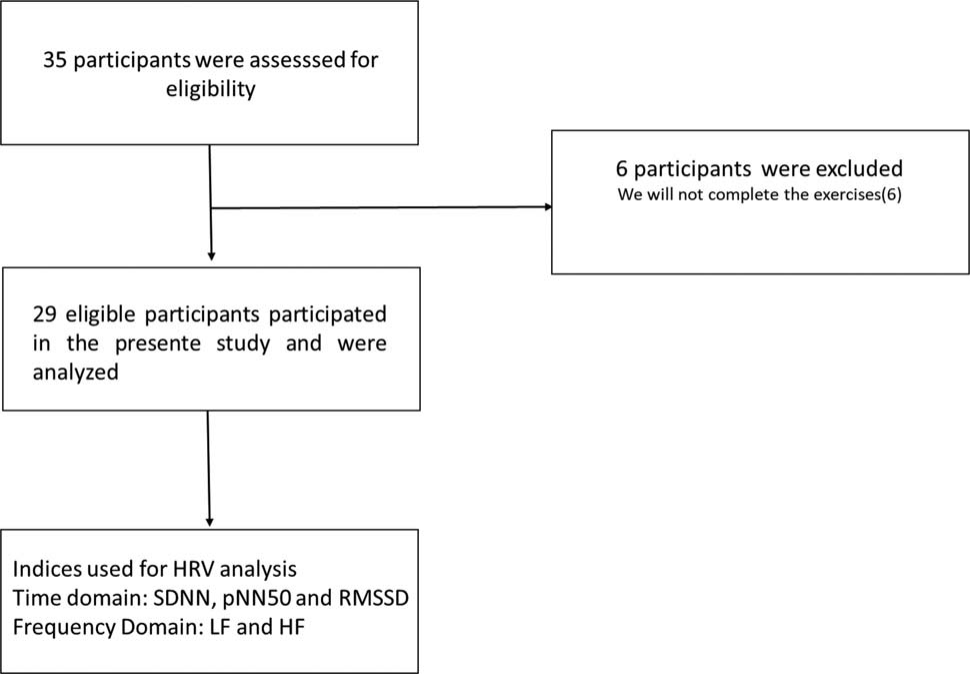
Flowchart of sample recruitment. HF = high frequency, HRV = heart rate variability, LF = low frequency, pNN50 = percentage of adjacent RR intervals with a duration difference greater than 50 ms, RMSSD = square root of the mean of the square of the differences between intervals, SDNN = standard deviation of all normal RR intervals.

Participants with cardiorespiratory, neurological, musculoskeletal disorders of the upper or lower limbs, and other known impairments that prevented the individual from performing the procedures were excluded from the study as well as those who failed complete the exercises (Fig. 1).

### 2.2 Data collection

The following information was collected: age, sex, weight, height, and body mass index (BMI). BMI was calculated using the following formula: body mass (kg)/height (m^2^).

### 2.3 Heart rate variability

Only series with more than 256 RR intervals were used in the analysis of HRV indices. In these series, digital and manual filtering was performed to eliminate artifacts and premature ectopic beats.

For the analysis of HRV in the frequency domain, the spectral components of low frequency (LF: 0.04–015 Hz) and high frequency (HF: 0.15–0.40 Hz) in ms^2^ and normalized units were used.

Time domain analysis was performed using standard deviation of the mean of normal RR intervals, percentage of adjacent RR intervals with a duration difference greater than 50 ms, and square root of the mean square of the differences between adjacent normal RR intervals.

RR intervals were recorded on a portable heart rate monitor RS800CX (Polar Electro, Kempele, Finland), a validated portable device with a sampling rate of 1 kHz). Data were analyzed using Polar Precision Performance software (Polar Electro), and a series with more than 95% heart rate were included. Then, it was exported in “txt” for final analysis in the Kubios HRV analysis program, version 2.1. Data analysis followed the guidelines of the Task Force.^[16,17]^

For HRV data collection, the volunteers remained seated at rest for 10 minutes. After the rest period, the participants performed the exercises. Immediately after the exercise protocol, the subjects remained seated at rest for 30 minutes. HRV indices were analyzed in the following periods: rest, 0 to 10 minutes, 10 to 20 minutes, and 20 to 30 minutes.

It was recommended to wear comfortable clothes and abstain from alcoholic or stimulant beverages within 24 hours before the tests, as well as having a light meal 2 hours before the tests and not exercising intensely the day before.

Data collection was carried out in a room with a temperature between 21°C and 25°C and humidity between 50 and 60%. The collection was carried out individually, from 6 AM to 9 AM to standardize the interferences of the circadian rhythm, and the volunteers were instructed to remain at rest.

The study participants were evaluated on 2 different days with approximately 48 hours of rest, alternating an exercise session for the upper limbs and another for the lower limbs. The order of the exercise protocols was randomized in relation to the body segment to be trained in each session. During the training of the electrocardiographic signal, the participants were instructed not to speak unnecessarily.

### 2.4 Blood pressure measurements

Systolic blood pressure and diastolic blood pressure were measured using a standard manual sphygmomanometer. Participants were strictly prohibited from consuming caffeinated products, smoking, and exercising 30 minutes before blood pressure measurement. Throughout the assessment, the patients remained in a relaxed sitting state for 5 minutes. The cuff was placed on the left arm for all patients, and the blood pressure was recorded according to standard guidelines. Blood pressure was measured in the following periods: rest, immediately after exercise, and 30 minutes after exercise by a health professional with skill in handling the equipment and not participating in the study.

### 2.5 Training protocol

The maximum load was determined from the maximum number of repetitions with submaximal loads. By multiplying by 2% by the number of repetitions performed, a percentage decrease in relation to 1 MR was found. The load used for a given number of repetitions corresponds to the percentage loss in relation to the maximum load, according to the formula: 1 MR = load ÷ [100% − (repetitions × 2)].^[18]^

The participants initially performed a general warm-up for 3 to 5 minutes. Subsequently, the maximum number of repetitions until exhaustion was verified with the intensity outlined for the session, with no pauses between the concentric and eccentric phases and between the repetitions. The test was completed when the participant was unable to perform the movement according to the evaluator's previous explanation. The results were then applied to the above formula.

The research participants performed 3 sets of 15 repetitions, estimating a 40% load of 1 MR in each exercise session and a resting time of one and min between sets.

The resistance exercise protocol for the upper limbs included barbell curls, French triceps, and lateral elevation of the upper limbs. For the lower limbs, plantar flexion, knee flexion, and knee extension exercises were used. The exercises were performed as described by Uchida et al.^[19]^

French triceps: sitting or standing, keeping the arm that is performing the exercise elevated (upright). Allow the forearm with the dumbbell to descend in a controlled manner until it forms an angle of less than 90° at the elbow joint, at which point the elbow should be extended. Return to the starting position and, without rest, repeat the movement; direct rose: stand upright, with the lower limbs laterally apart. Knees slightly bent, hand spread (approximately shoulder-width apart), and supination grip. Flex the elbows to the limit, return to the starting position and, without rest, repeat the movement; lateral elevation: Stand upright with your knees slightly bent, arms at your sides, and a dumbbell in each hand. From this position, shoulder abduction is performed until it reaches the horizontal position. Return to the starting position and, without rest, repeat the movement; Knee extension: Positioned on the device, with the back well supported and with the axis of the equipment very close to the axis of the knee joints, carry out the extension of the knees, using all possible range. Return to form a 90° angle at the knee joints and, without rest, repeat the movement; Knee flexion: Positioned on the device in the prone position, with the axis of the equipment very close to the axis of the knee joints, bend the knees until forming an angle of less than 90° in the knee joints. Return until the knees are practically extended and, without rest, repeat the movement; plantar flexion: position yourself in a standing position, with the device pad resting on your shoulders, spine erect, hip and knee joints extended, and hands resting on the device support to stabilize the movement. The ankle was bent until the heel was below the toe line. Raise the trunk as much as possible, slowly return to the starting position, and, without rest, repeat the movement.

### 2.6 Statistical analysis

Quantitative data are presented as measures of central tendency. Regarding HRV, the normality of the data was initially determined using the Shapiro–Wilk test. The Analysis of variance test was used, followed by Bonferroni posthoc test. In situations where the normal distribution was not accepted, the Friedman test followed by Dunn post-test was used. Differences in these tests were considered statistically significant when the *P*-value was less than .05. The statistical program used was Biostat 2009 Professional 5.8.4 (Analystsoftware, California). The sample size was based on a prior studies,^[11,12]^ when the significance level was set at 0.05, a sample size of 29 has 80.0% power to detect an effect size.

## 3 Results

Table 1 shows the characteristics of the sample, including age, height, weight, BMI, education, smoking, diseases, use of alcohol, and medication.

**Table 1:** General characteristics of the elderly participants in the study (n = 29).

Characteristics	Values
Age (yr)	65.37 ± 3.74
Height (m), mean (SD)	1.53 (0.07)
Weight (kg), mean (SD)	66.13 (10.63)
BMI, mean (SD)	28.24 (5.58)
Education	
Incomplete elementary education, %	20.68
Complete elementary education, %	6.89
Complete high school, %	27.58
Incomplete high school, %	3.48
Higher education, %	41.37
Smoking	
Yes, %	3.44
No, %	96.56
Alcoholic beverage	
Yes, %	3.44
No, %	96.56
Use of medicines	
Yes, %	79.31
No, %	20.69
Beta blockers, %	37.93
Antiarrhythmics, %	3.44
Others, %	37.93
Diseases	
Diabetes, %	13.79
Hypertension, %	34.48
Heart attack, %	3.44
Allergy, %	3.44
Osteoporosis, %	6.89

BMI = body mass index, SD = standard deviation, m = meters, kg = kilograms.

Table 2 shows the analysis of the HRV from the indexes related to the time and frequency domains in the exercise protocols performed for the upper limbs in the evaluated elderly.

**Table 2:** Analysis of HRV according to moments and type of exercise for upper limbs in elderly women, 2020.

Variables	Moment 1	Moment 2	Moment 3	Moment 4	*P*
Mean RR	821.9 (776.85; 861.02)	778 (730.44; 810.14)	786.5 (747.02; 839.76)	799.6 (755.35; 839.07)	.633
Mean HR	73.09 (69.78; 76.04)	77.78 (74.39; 82.80)	76.37 (71.52; 80.43)	75.62 (72.92; 79.49)	.417
SDNN	38.5 (31.93; 42.42)	49.3 (38.35; 59.78)^∗^	31 (27.76; 36.32)	32.1 (26.82; 40.08)	.938
RMSSD	22.1 (12.63; 34.71)	80.76 (12.42; 28.79)^∗^	16.8 (10.67; 23.33)	19.6 (11.62; 26.46)	.802
pNN50	2.7 (0.3; 9.78)	1.2 (0.4; 5.75)	0.9 (0.2; 3.93)^†^	1.2 (0.2; 4.93)	.701
LF (ms^2^)	219 (145.93; 372.79)	224 (144.63; 382.12)^∗^	182 (114.63; 321.99)^†^	218 (151.85; 452.39)	.553
LF (n.u)	70.6 (52.11; 78.15)	76.8 (60.71; 81.87)	75.8 (58.60; 82.22)	75.1 (59.71; 80.62)	.263
HF (ms^2^)	83 (41.24; 296.88)	102 (32; 185)^∗^	75 (28.69; 163.30)^†^	95 (39.77; 159.20)	.705
HF (n.u)	29.2 (21.77; 47.61)	23.2 (18.09; 39.12)	24.1 (17.70; 41.22)^†^	24.7 (19.30; 40.22)	.863
LF/HF	2.41 (1.09; 3.58)	3.31 (1.57; 4.52)	3.14 (1.42; 4.64)	3.03 (1.48; 4.18)	.263

Friedman test, posthoc Dunn posthoc test.

∗ significance with moment 1.

† significance with moment 2. 95% CI = 95% confidence interval, HF = high frequency, LF = low frequency, Mean HR = mean heart rate, MeanRR = RR intervals mean, RMSSD = square root of the mean of the square of the differences between intervals, s.u = standardized unit, SDNN = standard deviation of all normal RR intervals.

Table 3 shows the analysis of the HRV from the indices related to the time and frequency domains in the exercise protocols performed for lower limbs in the elderly evaluated.

**Table 3:** Analysis of HRV according to moments and type of exercise for the lower limb in elderly women, 2020.

Variables	Moment 1	Moment 2	Moment 3	Moment 4	*P*
Mean RR	758.5 (742.01; 862.22)	730.9 (703.00; 792.58)	775.8 (742.87; 863.86)	794.6 (772.57; 846.36)	.299
Mean HR	79.32 (69.86; 80.96)	82.41 (73.76; 85.13)	77.87 (69.77; 80.93)	75.63 (71.11; 77.79)	.811
SDNN	33.5 (29.76;42.03)	43 (37.39; 47.03)	31.3 (27.88; 40.64)	36.3 (29.19; 41.98)	.826
RMSSD	15.5 (11.70; 36.67)	13.9 (11.17; 29.25)	21.7 (12.23; 32.24)^†^	16.4 (12.03; 27.73)	.633
pNN50	0.7 (0.2; 8.31)	0.6 (0.26; 5.63)	0.6 (0.1; 6.41)	0.9 (0.23; 5.38)^‡^	.602
LF (ms^2^)	218 (142.32; 349.75)	207 (155.54; 307.53)	174 (118; 313.34)^†^	191 (124.77; 358.20)	.553
LF (n.u)	65.8 (56.78; 77.28)	75.2 (67.44; 81.75)	64.1 (52.37; 76.07)	62.8 (57.10; 76.18)	.263
HF (ms^2^)	76 (45.08; 265.05)	46 (35; 217.73)^∗^	65 (42.46; 254.14)^†^	76 (40.32; 240.12)	.705
HF (n.u)	34.1 (22.31; 45.48)	24.8 (18.04; 32.00)^∗^	35.1 (23.73; 47.55)	37.1 (23.75; 42.86)	.863
LF/HF	1.92 (1.32; 3.48)	3.03 (4.52; 4.52)	1.82 (1.11; 3.22)^†^	1.69 (1.33; 3.21)	.721

Friedman test, posthoc Dunn test.

∗ Significance with moment 1.

† Significance at moment 2.

‡ Significance with moment 3. 95% CI = 95% confidence interval, HF = high frequency, LF = low frequency, Mean HR = mean heart rate, MeanRR = RR intervals mean, RMSSD = square root of the mean of the square of the differences between intervals, s.u = standardized unit, SDNN = standard deviation of all normal RR intervals.

Regarding the mean RR intervals and heart rate surveyed, no statistically significant differences were found in the protocols for the upper and lower limbs in relation to these 2 variables at the time of analysis.

Regarding the indices of the time domain (standard deviation of all normal RR intervals, square root of the mean square of the differences between adjacent normal RR intervals, and percentage of adjacent RR intervals with a duration difference greater than 50 ms) and frequency domain (low frequency and high frequency), there were also no statistically significant differences between the 4 moments surveyed in the protocols for upper and lower limbs.

Table 4 shows the behavior of blood pressure among the volunteers surveyed at 3 different times (rest, immediately after exercise, and 30 minutes after exercise) in the exercise protocols for upper and lower limbs. Therefore, it was verified that no significant differences were found in systolic and diastolic pressures between the 3 moments surveyed in the protocols for the upper and lower limbs.

**Table 4:** Analysis of blood pressure according to moments and type of exercise in elderly women, 2020.

Variables	Moment 1	Moment 2	Moment 3	*P*
Upper limb exercise				
SBP (mm Hg)^∗^	128 (119.69; 139.30)^†^	134 (125; 146.91)	127 (118.77; 135)	.805
DBP (mm Hg)^∗^	77 (72; 76.04)	74 (69.69; 83.61)	74 (69.69; 80.30)	.691
Lower limb exercise				
SBP (mm Hg)^∗^	131 (124; 137.72)	138 (127.83; 147.72)	127.5 (114.63; 321.99)	.675
DBP (mm Hg)^∗^	79.5 (74.55; 81.72)	81 (73.27; 85.16)	75.5 (72.27; 79.72)^‡^	.863

Friedman test, posthoc Dunn test – ^†^significance with moment 0; ^‡^significance with moment 1; ^§^significance with moment 2. DBP = diastolic blood pressure, SBP = systolic blood pressure.

∗ Values expressed as median (95% confidence interval).

## 4 Discussion

This study evaluated the effect of resistance exercise on cardiac autonomic modulation in elderly women. There were no changes in the behavior of cardiac autonomic modulation and blood pressure between recovery times and after resistance exercises.

Aerobic training performed regularly is shown to be an effective nonpharmacological means to minimize the deleterious consequences of aging on the elderly; however, only resistance exercise reduces the effects on the muscular system.^[20,21]^

Previous studies have shown that there is an interaction between cardiac autonomic regulation and muscle stimuli. Body positioning influences individual muscle activity and the ratio of muscles. The pressor reflex to exercise is a peripheral neural reflex that originates in skeletal muscle. This reflex significantly contributes to the regulation of the cardiovascular system during exercise. The afferent arm of this reflex is composed of afferent fibers sensitive to metabolic stimuli and afferent fibers that are sensitive to mechanical stimuli. In this sense, the HRV records the time oscillations between 2 consecutive R waves, called RR interval, influenced by the sympathetic nervous system and parasympathetic nervous system over the sinoatrial node.^[9,16,17]^

Aging is accompanied by a progressive decline in muscle mass and muscle strength. Physical training, especially resistance exercise, has been shown to be an efficient strategy for preventing muscle atrophy, promoting muscle growth, and maintaining muscle function with advancing age. Studies have pointed to a positive association between sarcopenia and falls and fractures in the elderly. Sarcopenia is associated with reduced mobility, impaired upright balance, functional decline, hospitalization, and mortality.^[6,22]^

Aging is associated with a decline in cardiovascular function and changes in the structure and function of the autonomic nervous system. Studies on the effect of resistance exercise on cardiac autonomic modulation in the elderly are still scarce, with different training protocols in the literature with consequent different responses to exercise. In the present study, by adopting a 40% load of 1 MR in the exercise protocols for upper and lower limbs, no significant differences were found in the indexes of the HRV time and frequency domains between the 4 research periods (rest, immediately after exercise, and 20 and 30 minutes after exercise). The hypothesis regarding this behavior of HRV variables may be associated with factors such as training volume, intensity, influence of antihypertensive drugs, and decreased chronotropic responses associated with advancing age.^[23,24]^

The magnitude of the autonomic, cardiopulmonary, and metabolic adaptations generated by the practice of physical exercises to supply the muscular metabolic demand depends on the type, intensity, and duration of the activity, thus explaining the differences in relation to the autonomic behavior from the different stimuli used. Some authors^[23]^ observed a significant increase in systolic blood pressure after exercise performed at 80% of 1 MR, but not after exercise performed at 50% of 1 MR, pointing to an influence of exercise intensity with repercussions on hemodynamic factors. Maior et al,^[25]^ when verifying the acute effects of different intensities of resistance exercise (6 vs 12 maximum repetitions – 1 MR) on cardiac autonomic behavior during strength training composed of 6 exercises, did not observe statistical differences between the groups researched in none of the domains surveyed. However, in the intragroup comparison (pre vs post), they observed differences in the diverse intensities both in the time domain and in the frequency-related indices, suggesting the influence of the magnitude of the cardiac autonomic impact caused by training with high intensity and high volume, promoting reduction of vagal activity in the sinus node through the response of the central command, possibly due to ischemic behavior. The literature describes that resistance exercise influences the cardiovascular system, triggering several autonomic adjustments to supply the increase in local blood flow due to the metabolic increase caused by the physical activity in question.^[25]^ In static resistance exercises marked by mechanical obstruction of blood flow, the cardiovascular response to exercise depends on the intensity, duration, and muscle mass involved in the physical activity in question. In dynamic resistance exercises, there is no mechanical obstruction of blood flow, with an increase in sympathetic activity, with a consequent increase in heart rate, stroke volume, and cardiac output. The production of metabolites leads to a dilation process of the worked muscles, promoting a reduction in peripheral resistance, which is expressed through the reduction of diastolic blood pressure. ER also decreases plasma volume, promoting a hypotensive effect.^[24–27]^

However, the decrease in the response capacity of the baroreceptor mechanisms caused by aging can lead to inconsistent results. Dynamic resistance exercise can cause a smaller change in heart rate in the elderly than in young people. This reduced chronotropic response can be attributed to changes in cardiac autonomic function associated with decreased binding of adrenergic receptors and reduced activity of postreceptor proteins, factors related to increased heart rate in young people, and impaired in elderly subjects because of the decreased influence vagal over the heart.^[17]^

Another factor that may have influenced the results obtained in this study was the use of medicines by most of the elderly women evaluated. Of those surveyed, 79.31% were confessed to using medication. Carpio-Rivera et al^[28]^ studied the acute effect of exercise on blood pressure and observed an inverse correlation between blood pressure and age, body mass, duration of the training session, and number of sets, also verifying a greater hypotensive effect in physically active individuals who did not take antihypertensive medication.

Although resistance exercise is recommended for the elderly, changes resulting from these activities in relation to the intense elevations in blood pressure and cardiac autonomic imbalances can lead to cardiovascular risks. Exercise protocols that induce less pronounced autonomic responses are considered safe.^[27]^

The present study presented as a limitation the use of only 3 exercises per session for upper and lower limbs, differing from the training sessions that use a greater volume of training with a greater diversity of physical exercises.

## 5 Conclusion

Resistance exercises performed with low-intensity loads and a greater number of repetitions did not promote variation in the autonomic modulation of heart rate and blood pressure levels, showing good safety in elderly women.

## Acknowledgments

We thank the ABC Medical School and Regional University of Cariri for their support.

## Author contributions

**Conceptualization:** Rodrigo Daminello Raimundo, Naerton José Xavier Isidoro, Fernando Rocha Oliveira

**Data curation:** Rodrigo Daminello Raimundo, Naerton José Xavier Isidoro

**Formal analysis:** Fernando Rocha Oliveira

**Investigation:** Rodrigo Daminello Raimundo, Naerton José Xavier Isidoro

**Methodology:** Rodrigo Daminello Raimundo, Naerton José Xavier Isidoro

**Project administration:** Rodrigo Daminello Raimundo

**Resources:** Rodrigo Daminello Raimundo, Naerton José Xavier Isidoro, Fernando Rocha Oliveira

**Supervision:** Rodrigo Daminello Raimundo

**Validation:** Rodrigo Daminello Raimundo, Fernando Rocha Oliveira

**Visualization:** Rodrigo Daminello Raimundo, Fernando Rocha Oliveira

**Writing – original draft:** Rodrigo Daminello Raimundo, Naerton José Xavier Isidoro, Fernando Rocha Oliveira

**Writing – review & editing:** Rodrigo Daminello Raimundo, Naerton José Xavier Isidoro; Fernando Rocha Oliveira
